# Supplementary material for: Novel Antiviral Molecules against Ebola Virus Infection
Source: Int J Mol Sci. 2023 Sep 30;24(19):14791. doi: 10.3390/ijms241914791 (PMC10573436; doi:10.3390/ijms241914791)
Supplement: Supplementary file 1 [file ijms-24-14791-s001.zip › ijms-2611142-supplementary.pdf]

International Journal of
Molecular Sciences

MDPI

Novel Antiviral Molecules against Ebola Virus Infection

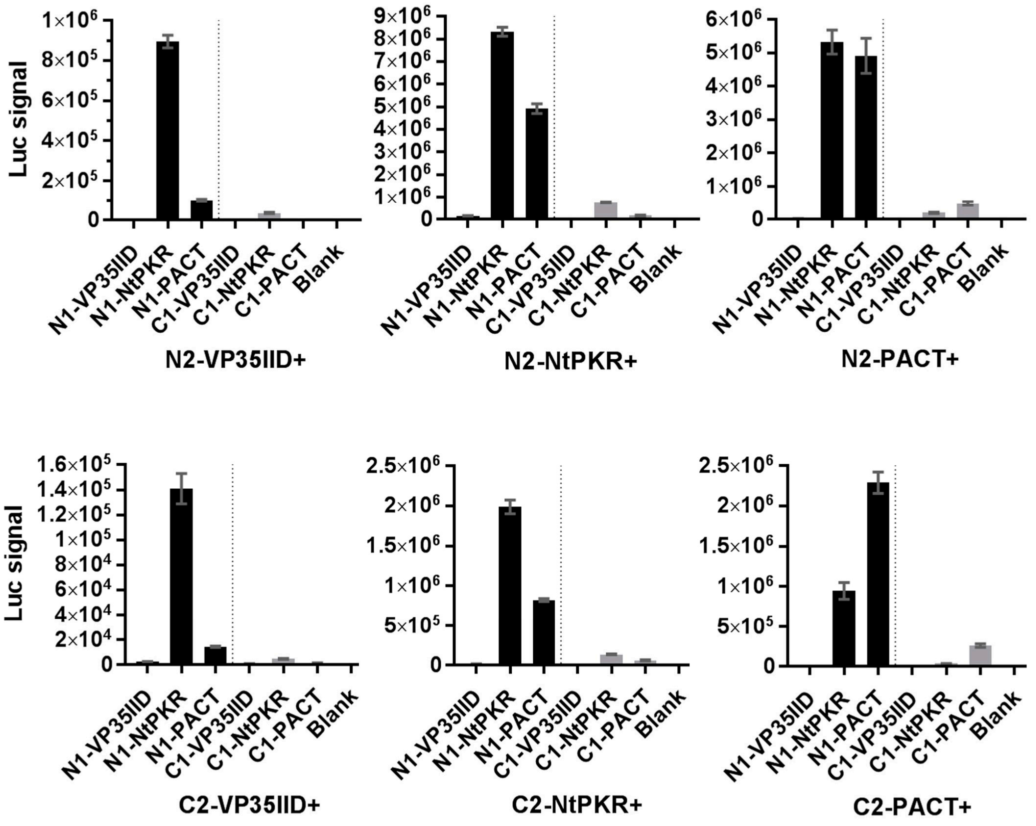

Figure S1. Interaction of VP35IID with NtPKR or PACT *in cellula*. NPCA of plasmids expressing the nanoluciferase N1 and N2 moieties fused to the N- or C-terminus of VP35IID, NtPKR or PACT in all possible combinations. Representative graphs are shown.

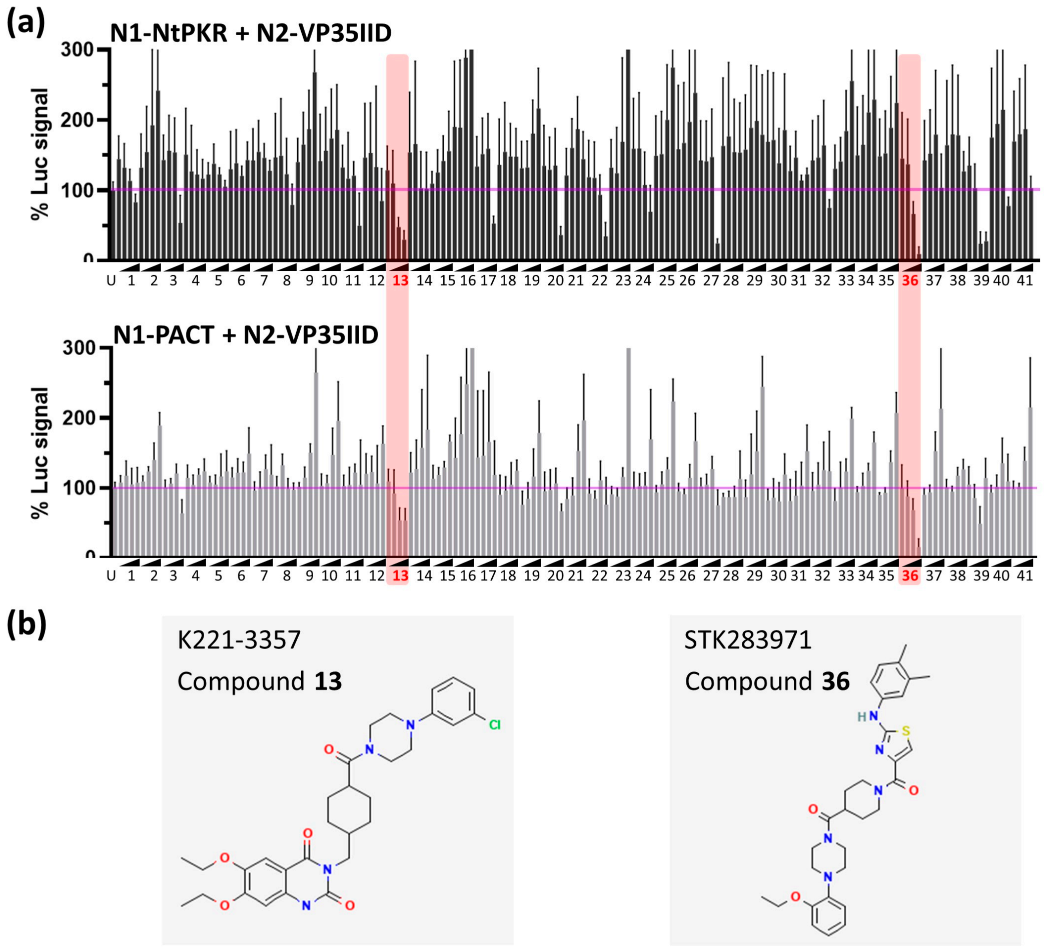

Figure S2. Highlights of compounds **13** and **36**. (**a**) Luciferase signal (measured for 100 ms) recorded 24 h after the 41 compounds at 1, 5, 10 and 20 µM or equivalent concentrations of DMSO (not shown) were applied to HEK293T cells transfected with either the mix N1-NtPKR/N2-VP35IID (upper graph) or N1-PACT/N2-VP35IID (lower graph). U, untreated with DMSO or compounds used to normalize luciferase signals from three independent experiments in technical triplicates; the horizontal purple line indicates the 100% signal. (**b**) Chemical 2D structure of compounds **13** and **36**.

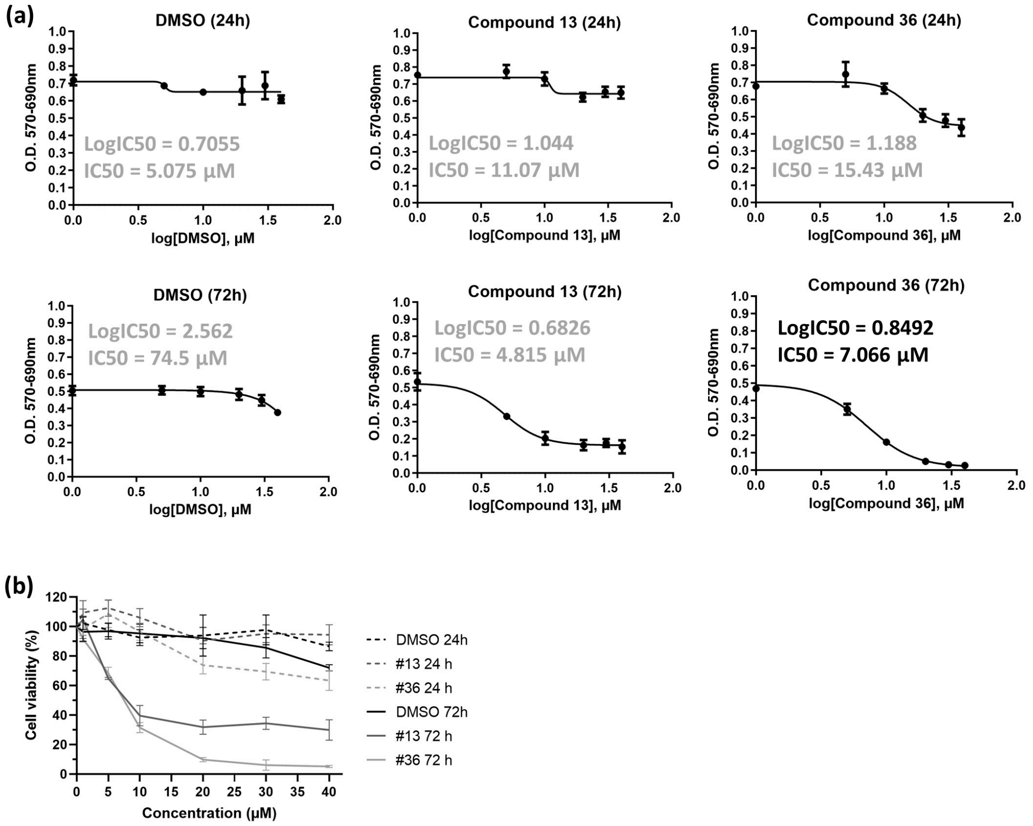

Figure S3. Compounds **13** and **36** additional cytotoxicity assessment in Huh7.25-CD81 cells. MTT assay was used to estimate IC_{50} (**a**) and % of cell viability (**b**). Please, note that only IC_{50} of compound **36** at 72 h post treatment may be correctly estimated since O.D. did not reach 0 in any other case even upon treatment at 40 µM for 72 h.

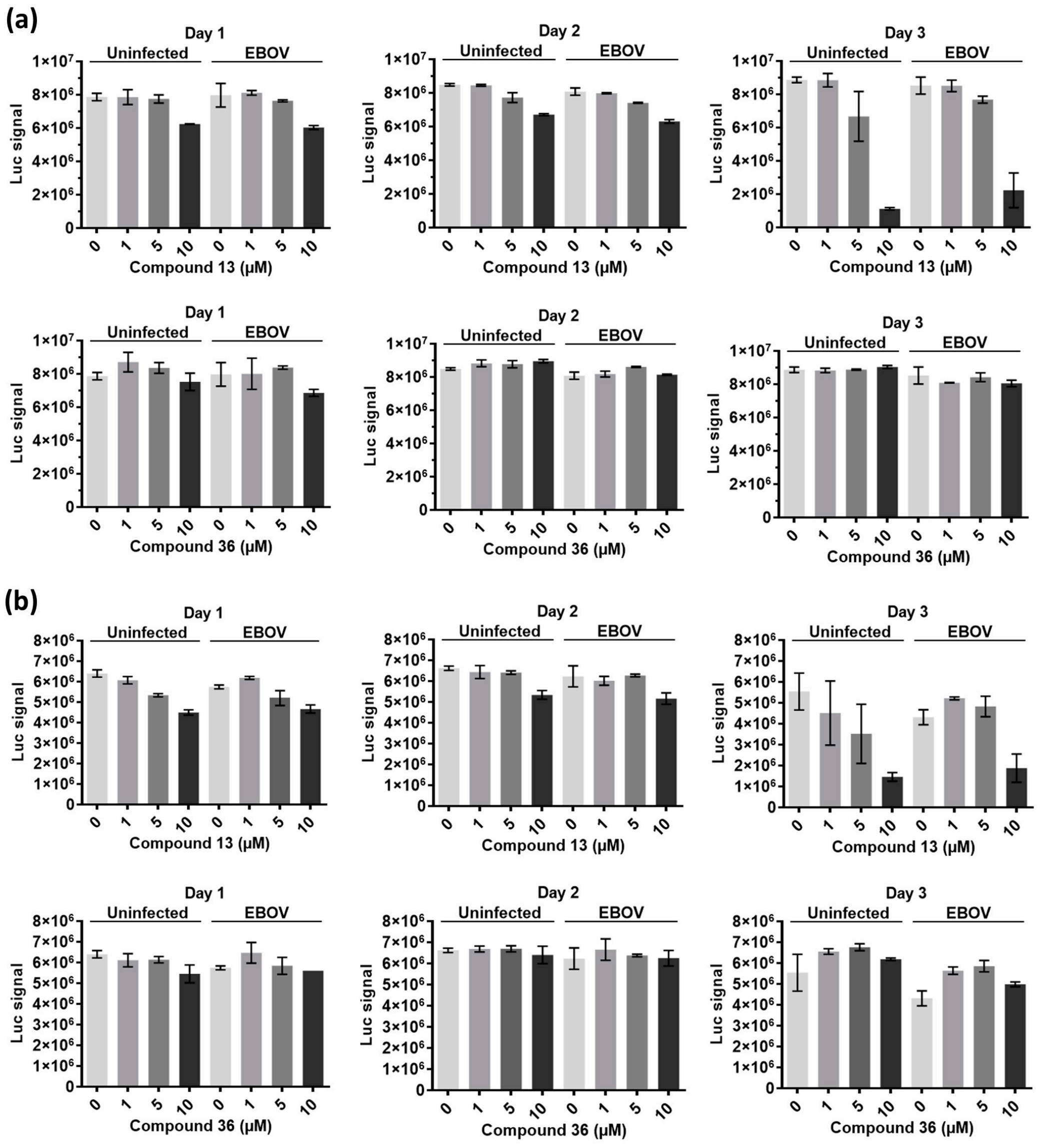

Figure S4. Cell viability assay corresponding to Figure 3. The viability of 293T cells (**a**) and Huh7 cells (**b**) treated with the compounds **13** or **36**, with or without EBOV infection was quantified as luciferase signal (Luc signal) at 24, 48, and 72 h post-infection (Day1, Day 2, Day 3, respectively) using the CellTiter-Glo 2.0 assay (Promega, #G9681) following the manufacturer's instructions.

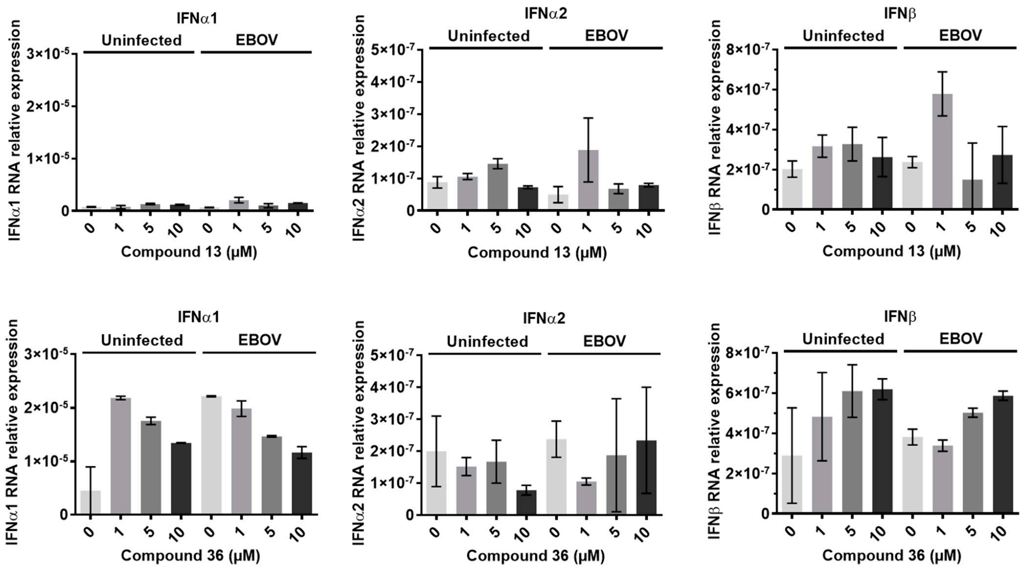

Figure S5. Effect of compounds **13** and **36** on IFN induction during EBOV infection. RNA extracted from EBOV-infected HEK293T (same samples as in Figure 3) were analyzed for expression of the two early (IFNβ, IFNα1) and one late (IFNα2) genes by RT-qPCR.

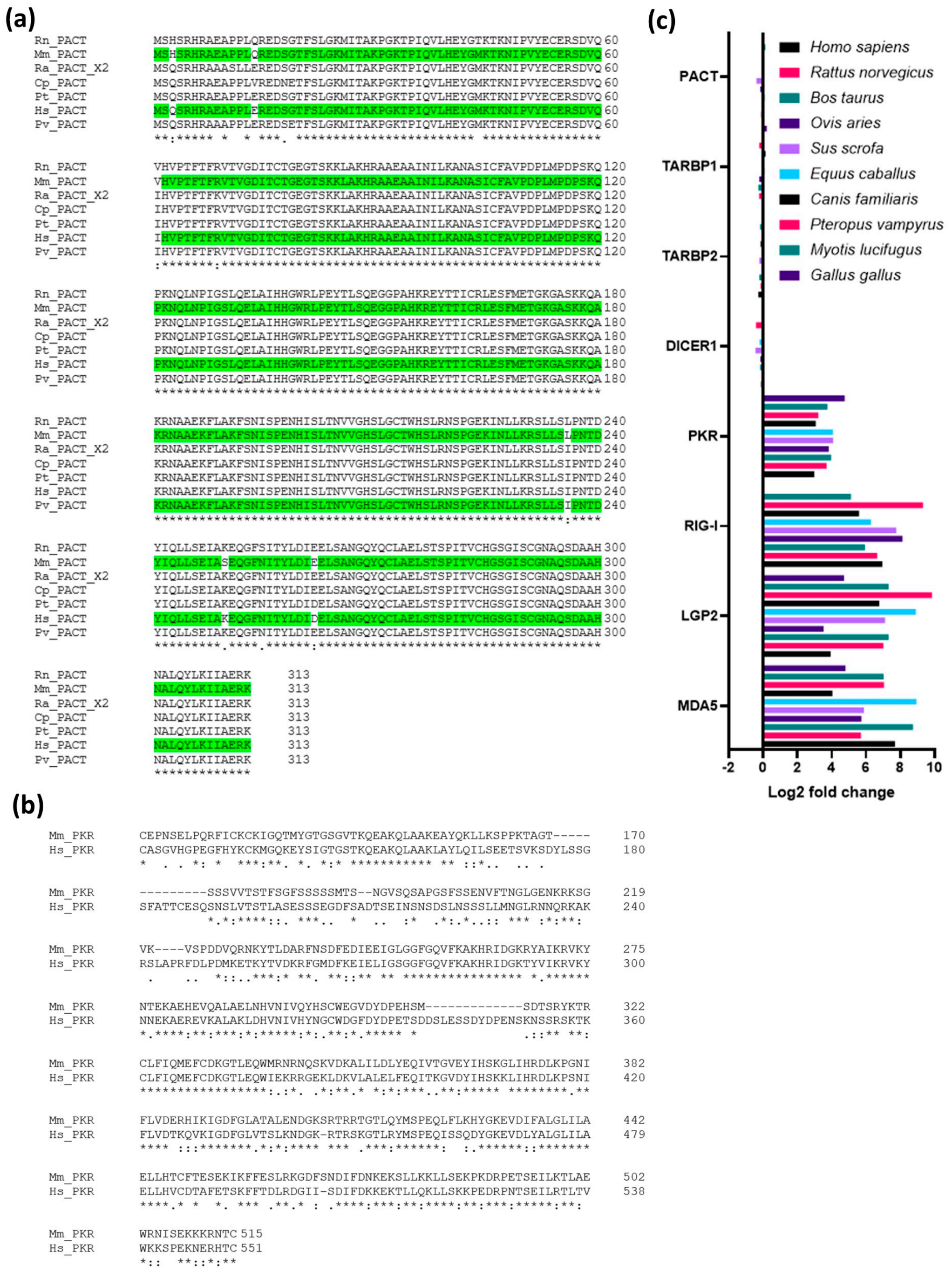

Figure S6. PACT and PKR protein alignment and expression upon type I IFN. Clustal Omega Multiple Sequence Alignment (https://www.ebi.ac.uk/Tools/msa/clustalo/) [1] was used to align PACT/PRKRA Uniprot or NCBI Reference Sequences of rat *Rattus norvegicus* (Rn, Q4V8C7), mouse *Mus musculus* (Mm, Q9WTX2), Egyptian fruit bat *Rousettus aegyptiacus* (Ra, NCBI: XP_016008061.1), Guinea pig *Cavia porcellus* (Cp, H0V1X9), chimpanzee *Pan troglodytes* (Pt, A0A6D2WC05), human *Homo sapiens* (Hs, O75569) and flying fox *Pteropus vampyrus* (Pv, A0A6P3Q6R0) in (**a**), and PKR/E2AK2 of human (P19525) and mouse (Q03963) in (**b**). (**c**) Lack of PACT induction upon type I IFN treatment across the indicated vertebrate species; data retrieved from (http://isg.data.cvr.ac.uk/) [2] evidencing that PACT (PRKRA) is not an interferon stimulated gene (ISG), similarly to the rest of RNAi pathway proteins (TARBP1/2 and DICER1), in contrast to de bona fide ISGs PKR (EIF2AK2), RIG-I (DDX58), LGP2 (DHX58) and MDA5 (IFIH1).

Table S1. Primers used in this study for cloning or for RTqPCR (in italics).

Name	Sequence (5′-3′)
VP35F	GCGATCGCCATGGGAAAGCCCGACATTAGTGCTAAG
VP35R	ACGCGTAATCTTCAGACCCAGGGTCTTGCC
VP35-B1	GGGGACAACTTTGTACAAAAAAGTTGGCATGGGAAAGCCCGACATTAGTGCTAAG
VP35-B2N	GGGGACAACTTTGTACAAGAAAGTTGGAATCTTCAGACCCAGGGTCTTGCCG
VP35-B2S	GGGGACAACTTTGTACAAGAAAGTTGGTTAAATCTTCAGACCCAGGGTCTTGCCG
M1-F	CCCTCCCTCCCCAGCCATCGACGCCGGCTGGGTCTGCG
M1-R	CGCAGACCCAGCCGGCGTCGATGGCTGGGGAGGGAGGG
M2-F	TGCCAGAAGTCCCTGGCCCCAGTCCCTCCCTC
M2-R	GAGGGAGGGACTGGGGCCAGGGACTTCTGGCA
M3-F	CTTTGGCACCGCCGCCCACCAGCTGGTGCAG
M3-R	CTGCACCAGCTGGTGGGCGGCGGTGCCAAAG
M4-F	GGCGACATCCCCGCCGCTTGCCAGGCCTCCCTGCGACCAG
M4-R	CTGGTCGCAGGGAGGCCTGGCAAGCGGCGGGGATGTCGCC
M5-F	CCAGGGCTTGCCAGGCCTCCCTGGCCCCAGTCCCTCCC
M5-R	GGGAGGGACTGGGGCCAGGGAGGCCTGGCAAGCCCTGG
M6-F	ACATCCCCGCCGCTTGCCAGAAGTCCCTGGCCCCAGTCCC
M6-R	GGGACTGGGGCCAGGGACTTCTGGCAAGCGGCGGGGATGT
FB_PKR1	GGGGACAACTTTGTACAAAAAAGTTGGCATGGCTGGTGATCTTTCAGC
RB_PKR1	GGGGACAACTTTGTACAAGAAAGTT-<br>GGTTATAAAGGACTAACTGCCTTCTTTTCCTTA
FB_PKR2	GGGGACAACTTTGTACAAAAAAGTTGGCTCTTCAGAAGGATTATCCATGGG
RB_PKR2	GGGGACAACTTTGTACAAGAAAGTTGGTTAGTCAGATTTCACTGAGGTTTCTTCT
FB_PACT1	GGGGACAACTTTGTACAAAAAAGTTGGCATGATAACAGCTAAGCCAGGG
RB_PACT1	GGGGACAACTTTGTACAAGAAAGTTGGTTAAACTGCAAAGCAAATACTTGCA
FB_PACT2	GGGGACAACTTTGTACAAAAAAGTTGGCTCCAAGCAACCAAAGAACC
RB_PACT2	GGGGACAACTTTGTACAAGAAAGTTGGTTAAGAAATGTGGTTCTCTGGAGA
IFNα1-F	GTGGTGCTCAGCTGCAAGTC
IFNα1-R	TGTGGGTCTCAGGGAGATCAC
IFNα1 probe	AGCTGCTCTCTGGGC (FAM)
IFNα2-F	CAGTCTAGCAGCATCTGCAACAT
IFNα2-R	GGAGGGCCACCAGTAAAGC
IFNα2 probe	ACAATGGCCTTGACCTT (FAM)
IFNβ-F	TCTCCACGACAGCTCTTTCCA
IFNβ-R	ACACTGACAATTGCTGCTTCTTTG
IFNβ-probe	AACTTGCTTGGATTCCT (FAM)
IFNβ-F(MV)	AAGCAATTGTCCAGTCCCA
IFNβ-R(MV)	TGCATTACCTGAAGGCCAAG
MV-F	TCAGGCATACCCACTAGTGTGAA
MV-R	TGACAGATAGCGAGTCCATAACG
GAPDH-F	GGTCGGAGTCAACGGATTTG
GAPDH-R	ACTCCACGACGTACTCAGCG

1. Sievers, F.; Wilm, A.; Dineen, D.; Gibson, T.J.; Karplus, K.; Li, W.; Lopez, R.; McWilliam, H.; Remmert, M.; Soding, J.; et al. Fast, scalable generation of high-quality protein multiple sequence alignments using Clustal Omega. *Mol. Syst. Biol.* **2011**, *7*, 539. https://doi.org/10.1038/msb.2011.75.
2. Shaw, A.E.; Hughes, J.; Gu, Q.; Behdenna, A.; Singer, J.B.; Dennis, T.; Orton, R.J.; Varela, M.; Gifford, R.J.; Wilson, S.J.; et al. Fundamental properties of the mammalian innate immune system revealed by multispecies comparison of type I interferon responses. *PLoS Biol.* **2017**, *15*, e2004086. https://doi.org/10.1371/journal.pbio.2004086.
